# Crystal structure of sodium (1*S*)-d-mannit-1-yl­sulfonate

**DOI:** 10.1107/S2056989018011556

**Published:** 2018-08-24

**Authors:** Alan H. Haines, David L. Hughes

**Affiliations:** a School of Chemistry, University of East Anglia, Norwich, NR4 7TJ, UK

**Keywords:** crystal structure, d-mannose bis­ulfite adduct, sodium hydrogen sulfite, sodium metabisulfite

## Abstract

The title salt, Na^+^·C_6_H_13_O_9_S^−^ [systematic name: sodium (1*S*,2*S*,3*S*,4*R*,5*R*)-1,2,3,4,5,6-hexa­hydroxy­hexane-1-sulfonate], is formed by reaction of d-mannose with sodium bis­ulfite (sodium hydrogen sulfite) in water. The anion has an open-chain structure with the S atom and the C atoms of the carbohydrate chain forming an essentially planar zigzag chain in which the absolute values of the torsion angles lie between 173.6 (2) and 179.9 (3)°. The sodium cations are penta-­coordinated by O atoms, with one link to a carbohydrate O atom and four to O atoms of sulfonate residues in separate anions, thus creating a three-dimensional network. The carbohydrate anions are arranged in a head (–SO_3_^−^) to head (–SO_3_^−^) arrangement, thereby forming two parallel sheets linked through coordination to sodium ions, with each sheet containing inter­molecular hydrogen bonds between the anionic residues. Unusually, the double sheets are not connected to neighbouring sets of double sheets, either by ion coordination or inter­molecular hydrogen bonding.

## Abstract 2

The title salt, Na^+^·C_6_H_13_O_9_S^−^ [systematic name: sodium (1*S*,2*S*,3*S*,4*R*,5*R*)-1,2,3,4,5,6-hexa­hydroxy­hexane-1-sulfonate], is formed by reaction of d-mannose with sodium bis­ulfite (sodium hydrogen sulfite) in water. The carbohydrate anions are arranged in a head (–SO_3_^−^) to head (–SO_3_^−^) arrangement, thereby forming two parallel sheets linked through coordination to sodium ions, with each component sheet containing inter­molecular hydrogen bonds between the anionic residues. Unusually, the double sheets are not connected to neighbouring sets of double sheets, either by ion coordination or inter­molecular hydrogen bonding.

## Chemical context   

Adducts formed by the reaction of aldehydes and bis­ulfite anions have long been used for aldehyde purification since they are often crystalline, whereas the parent aldehydes are often liquids with varying stabilities on storage. The addition reaction is reversible, which makes the bis­ulfite compounds useful inter­mediates in the synthesis of other adducts from aldehydes, such as cyano­hydrins. Further, bis­ulfite adducts are useful since they are soluble in water, which can be important if the compounds need to be compatible with aqueous, biological systems, for example to aid delivery of medicinal drugs insoluble in water. Such considerations are important since aldehydes are involved in many synthetic processes for the production of commercially relevant compounds, including pharmaceuticals, and the relative advantages of using different counter-ions (*e.g.* stability, hygroscopicity, ease of filtration of the adduct) are of inter­est (Kissane *et al.*, 2013 ▸).

The bis­ulfite addition products of aldoses are unusual in that they are acyclic compounds despite the fact that the parent carbohydrates exist predominantly in the cyclic, hemi-acetal form. Although such adducts were synthesised many years ago, unequivocal proof of their acyclic nature awaited X-ray structure determination, firstly on the potassium adducts of d-glucose and d-mannose (Cole *et al.*, 2001 ▸), of d-galactose (Haines & Hughes, 2010 ▸), d-ribose (Haines & Hughes, 2014 ▸), d-lyxose (Haines & Hughes, 2015 ▸), and of the sodium adducts of d-glucose (Haines & Hughes, 2012 ▸) and d-lyxose (Haines & Hughes, 2016 ▸).

We now report the preparation, properties, and crystal structure of the sodium bis­ulfite adduct of d-mannose, and comment on its significant structural difference from that of the corresponding potassium adduct.
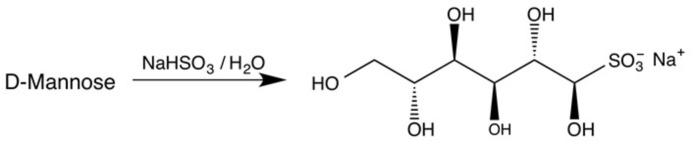


Mixing concentrated, equimolar solutions of d-mannose and sodium bis­ulfite (sodium hydrogen sulfite formed by the *in situ* hydrolysis of sodium metabisulfite) in water led to immediate precipitation of the adduct in high yield; this was purified by recrystallization from water, giving material stable in air but which melted over a large temperature range (413–444 K) with extended and continual decomposition.

Obtaining suitable crystals for X-ray analysis was challeng­ing since there was a tendency for formation of thin, rough, multiple crystals, but slow crystallization at approximately 283–286 K and careful selection from the crop so-produced afforded the crystal for examination. The newly formed chiral centre at C1 had the *S*-configuration and in solution in water:acetic acid (9:1) the adduct gave a positive rotation which remained stable over an extended period, suggesting that hydrolysis to its component parts was hindered under the acidic conditions.

## Structural commentary   

**Figure 1:**
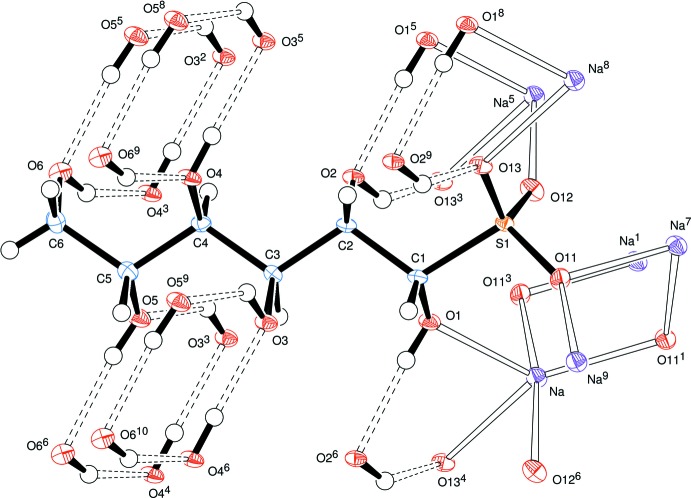
View of the d-mannose–NaHSO_3_ adduct, indicating the atom-numbering scheme. All sodium coordination contacts and hydrogen bonds involving the atoms of the sugar adduct are indicated. Displacement ellipsoids are drawn at the 50% probability level. Symmetry codes: (1) 1 − *x*, *y* − 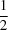, 2 − *z*; (2) 1 + *x*, *y* − 1, *z*; (3) *x*, *y* − 1, *z*; (4) *x* − 1, *y* − 1, *z*; (5) 1 + *x*, *y*, *z*; (6) *x* − 1, *y*, *z*; (7) 1 − *x*, 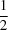 + *y*, 2 − *z*; (8) 1 + *x*, 1 + *y*, *z*; (9) *x*, 1 + *y*, *z*; (10) *x* − 1, 1 + *y*, *z*.

The newly formed chiral centre at C1 has the *S*-configuration (as shown in Fig. 1 ▸) and the systematic name for the salt is sodium (1*S*,2*S*,3*S*,4*R*,5*R*)-1,2,3,4,5,6-hexa­hydroxy­hexane-1-sulfonate. The anion has an open-chain structure in which the S atom and the C atoms of the sugar chain form an essentially planar zigzag (*all-trans*) chain with the corresponding torsion angles lying between the absolute values of 173.6 (2) and 179.9 (3)°. The hydrogen atoms of the hydroxyl groups on C1 to C6 of the carbon chain form hydrogen bonds with oxygen atoms O2, O13, O5, O3, O6 and O4, respectively, of neighbouring chains (Fig. 1 ▸ and Table 1 ▸) and all the hydroxyl O atoms except O1 are acceptors of hydrogen bonds; O1 is bonded to a sodium ion. We note that all the hydrogen bonds are arranged in cyclic systems, some comprising four O—H⋯O bonds, others with two O—H⋯O bonds plus two Na—O coordination bonds.

**Figure 2:**
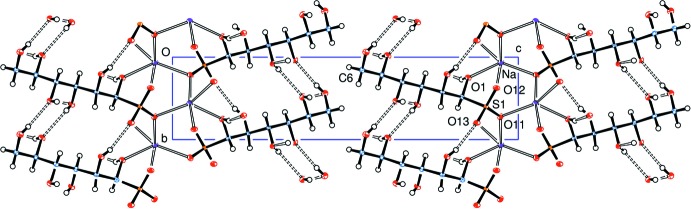
View down the *a* axis of the crystal packing.

Unusually, the sodium atom has a coordination sphere of five rather than six oxygen atoms, hexa-coordination having been observed in related adducts from d-glucose (Haines & Hughes, 2012 ▸) and d-lyxose (Haines & Hughes, 2016 ▸). Further, coordination of a sodium ion by O1 of the carbohydrate chain and oxygen atoms O11, O12 and O13 of four different sulfonate groups leads to a sheet of Na ions coord­inated to the ‘heads’ of the anions, Fig. 2 ▸.

The Na—O bonds have lengths in the range 2.293 (3) to 2.421 (3) Å (Table 2 ▸) and form a distorted square pyramidal shape with O1 in the apical site. The next shortest Na–O contact distance is 2.757 Å to O12^iii^, which would provide a rather distorted octa­hedral coordination. Twofold screw axes of symmetry (parallel to the *b* axis) relate the sodium ions in the sheet close to the *ab* plane at *z* = 0 and 1, and the zigzag *C*_6_ chains lie approximately normal to this plane and nearly parallel to the *c* axis. The ‘tails’ of these chains, around the C6,O6 groups, lie close to the *z* = 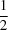 plane where the screw axes relate them to the tails of adjacent mol­ecules. But, whereas the heads are linked through the sodium atoms across the *z* = 0 plane, there are no short inter­molecular contacts across the *z* = 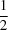 plane; the shortest contacts here are H61⋯H62^11^ = 2.57 Å, H61⋯O6^12^ = 2.70 Å, H62⋯H5^13^ = 2.60 Å and H62⋯C6^13^ = 2.95 Å, *i.e.* at normal van der Waals’ distances; symmetry codes: (11) 1 − *x*, *y* + 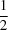, 1 − *z*; (12) 2 − *x*, *y* + 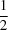, 1 − *z*; (13) 1 − *x*, *y* − 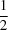, 1 − *z*.

**Figure 3:**
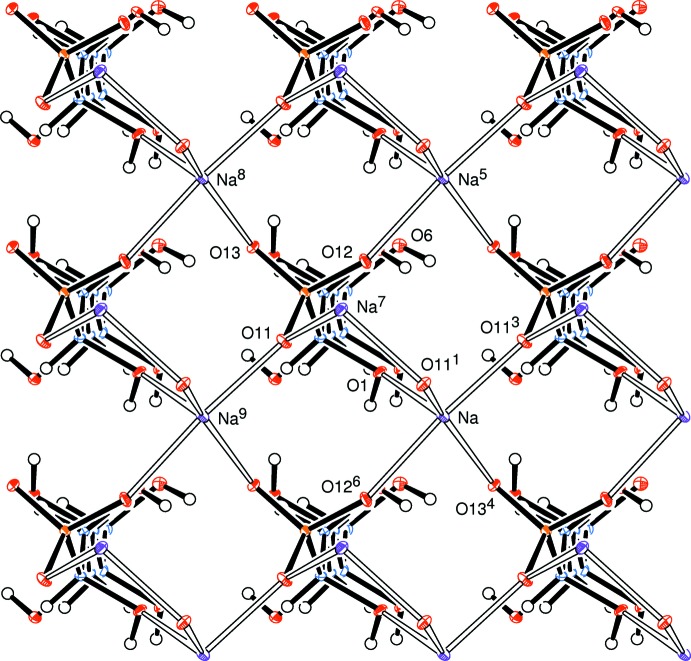
View looking along the all-*trans* sugar chain and neighbouring chains, all pointing in the same direction. Displacement ellipsoids are shown at the 30% probability level. Symmetry codes are defined as for Fig. 1 ▸.

**Figure 4:**
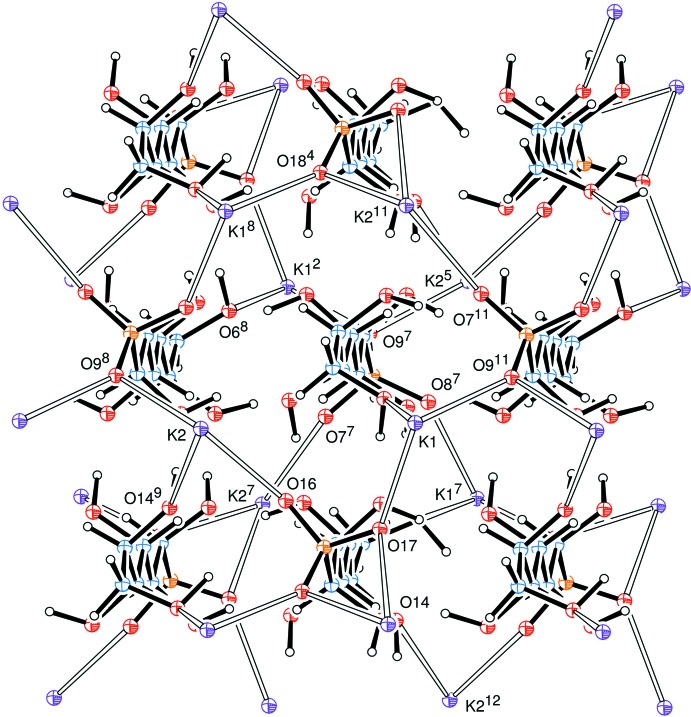
View looking along the all-*trans* sugar chain and neighbouring chains in the d-mannose–potassium bis­ulfite adduct; adjacent chains point in opposing directions. Atom coordinates were taken from the CCDC deposition, code 172060 (Cole *et al.*, 2001 ▸). Atoms are represented by small spheres of arbitrary radii. Symmetry codes: (2) 1 − *x*, 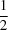 + *y*, −*z*; (4) 1 + *x*, *y*, *z*; (5) 1 − *x*, *y* − 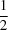, −*z*; (7) *x*, *y*, *z* − 1; (8) 1 − *x*, 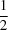 + *y*, 1 − *z*; (9) −*x*, 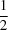 + *y*, −*z*; (11) 1 − *x*, *y* − 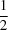, 1 − *z*; (12) −*x*, *y* − 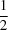, −*z*.

The neighbours of the zigzag *C*_6_ chains are related only by translation parallel to the *a* and *b* axes, Fig. 3 ▸; all the cations here are aligned in the same direction. Anions related across the Na coordination plane and about the *z* = 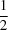 plane have the opposite alignment. Here, we observe a major difference between this sodium complex and the corresponding potassium d-mannose complex (Cole *et al.*, 2001 ▸) where each *C*_6_ chain is surrounded by four chains pointing in the opposite direction, as shown in Fig. 4 ▸. Hence the distances between the coordination planes are quite different, *viz* 21.08 Å in the sodium complex, but 11.55 Å for the potassium compound.

## Supra­molecular features   

A three-dimensional bonding network exists in the crystal structure through (i) penta-coordination of a sodium cation with oxygens from five different mannose bis­ulfite residues, and (ii) inter­molecular hydrogen bonds from each of the six hydroxyl groups to acceptor oxygen atoms in four different residues.

## Spectroscopic results   

High resolution mass spectrometry in negative ion mode showed, as the base peak in the spectrum, a peak for ([C_6_H_13_O_9_S_1_]^−^) at *m*/*z* 261.0287 and a significant peak was observed at *m*/*z* 243.0182 ([C_6_H_13_O_9_S_1_–H_2_O]^−^). A minor peak observed at *m*/*z* 359.1194 ([C_12_H_23_O_12_]^−^) was assigned to a dimer ion ([2*M* − H]^−^) produced by association of a d-mannose mol­ecule (*M* = C_6_H_12_O_6_) with the mono-anion of d-mannose ([C_6_H_11_O_6_]^−^) under the electrospray ionization conditions of the mass spectrometric measurement.

The ^1^H NMR spectrum of the adduct in D_2_O indicated the presence of the α- and β-pyran­ose forms of d-mannose and the major and minor forms of the acyclic sulfonate in the % ratios 25.24:13.14:55.00:6.62, respectively. Clearly, the *R*-stereo­isomer at C1 is present in solution but only the *S*-isomer crystallizes. Further, some hydrolysis of the adduct to afford the parent sugar occurs during the NMR measurement.

The ^13^C NMR spectrum showed signals for C1 nuclei at δ_C_ 94.70, 94.31, 84.43 and 82.34 arising, respectively, from the α- and β-pyran­ose forms of d-mannose, the minor adduct and the major adduct, in the % ratios of 32.73:15.00:3.64:48.63.

## Synthesis and crystallization   

d-Mannose (0.9 g) was dissolved in water (2 ml), sodium metabisulfite (0.475 g) was added, and the solution was then warmed to achieve complete solution. On cooling to room temperature, precipitation occurred within 3 min (see scheme[Chem scheme1]). The product was collected by filtration, and dried to give the adduct (1.42 g, 84%), a portion of which was recrystallized to afford the analytical sample, m.p. 413–444 K (with extended and continual decomposition); [α]_D_^20^ +8.2 (15 min.) (*c*, 0.79 in 9:1 H_2_O: HOAc). ^1^H NMR (D_2_O, 400 MHz, reference *Me_3_*COH at δ_H_ 1.24): δ_H_ 5.17 (*d, J*_1,2_ = 1.5 Hz, H-1 of α-pyran­ose), 4.89 (*d, J*_1,2_ = 0.8 Hz, H-1 of β-pyran­ose); signals for the major acyclic sulfonate: δ_H_ 4.64 (*s*, H-1), 4.20 (*d*, *J*_2,3_ = 9.5 Hz, H-2); for the minor acyclic sulfonate: δ_H_ 4.64 (*d, J*_1,2_ = 5.2 Hz, H-1), 4.07 (*d*, *J*_2,3_ = 7.8 Hz, H-2); ratio of major to minor sulfonate = 8.3:1. ^13^C NMR (D_2_O, 100 MHz, reference *Me_3_*COH at δ_C_ 30.29): δ_C_ 94.70 (C1, α-pyran­ose), 94.31 (C1, β-pyran­ose); signals for the major acyclic sulfonate: δ_C_ 82.34 (C1), 71.48, 69.46, 69.18*, 68.91* (C2, C3, C4, C5), 63.96 (C6); the minor acyclic sulfonate showed a peak at δ_C_ 84.43 (C1). Each of the signals marked with * is the average value of two closely spaced singlets of equal intensity separated by 4 Hz. The reasons for these small separations in the proton decoupled ^13^C spectrum are not clear.

Integration of the various signals for H-1 in the ^1^H NMR spectrum indicated that the species α-pyran­ose, β-pyran­ose, major acyclic sulfonate and minor acyclic sulfonate were present in the % ratios of 25.24: 13.14: 55.00: 6.62, respectively. In the ^13^C NMR spectrum, based on peak heights, the corresponding ratios were: 32.73: 15.00: 48.63: 3.64.

HRESMS (negative ion mode, measured in an H_2_O/MeOH, solution) gave a base peak at *m/z* 261.0287 ([C_6_H_13_O_9_S_1_]^−^), and a significant peak at 243.0182 ([C_6_H_13_O_9_S_1_–H_2_O]^−^).

## Refinement   

Crystal data, data collection and structure refinement details are summarized in Table 3 ▸. All the hydrogen atoms were located in difference maps and were refined with isotropic thermal parameters; the hydroxyl hydrogen atoms were refined with constrained O—H distances.

## Supplementary Material

Crystal structure: contains datablock(s) I. DOI: 10.1107/S2056989018011556/hb7768sup1.cif

Structure factors: contains datablock(s) I. DOI: 10.1107/S2056989018011556/hb7768Isup2.hkl

CCDC reference: 1862155

Additional supporting information:  crystallographic information; 3D view; checkCIF report

## Figures and Tables

**Table 1:** Hydrogen-bond geometry (Å, °)

*D*—H⋯*A*	*D*—H	H⋯*A*	*D*⋯*A*	*D*—H⋯*A*
O1—H1*O*⋯O2^ii^	0.81 (3)	1.92 (4)	2.693 (4)	160 (8)
O2—H2*O*⋯O1	0.81 (3)	2.36 (5)	2.828 (3)	118 (5)
O2—H2*O*⋯O13^i^	0.81 (3)	2.08 (4)	2.745 (4)	140 (5)
O3—H3*O*⋯O4	0.81 (3)	2.32 (7)	2.838 (3)	123 (7)
O3—H3*O*⋯O5^v^	0.81 (3)	2.11 (6)	2.743 (4)	136 (7)
O4—H4*O*⋯O3^vi^	0.82 (3)	1.87 (3)	2.692 (3)	179 (8)
O5—H5*O*⋯O6^ii^	0.80 (3)	1.91 (3)	2.704 (4)	169 (6)
O6—H6*O*⋯O4^i^	0.81 (3)	2.00 (4)	2.753 (4)	154 (6)
O6—H6*O*⋯O5	0.81 (3)	2.45 (6)	2.853 (4)	112 (5)

Symmetry codes: (i) 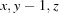; (ii) 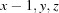; (v) 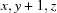; (vi) 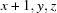.

**Table 2:** Selected bond lengths (Å)

Na—O11^i^	2.293 (3)	Na—O1	2.386 (3)
Na—O12^ii^	2.328 (3)	Na—O13^iv^	2.421 (3)
Na—O11^iii^	2.370 (3)	Na—O12^iii^	2.757 (3)

Symmetry codes: (i) 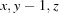; (ii) 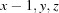; (iii) 
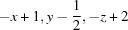
; (iv) 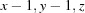.

**Table 3:** Experimental details

Crystal data	
Chemical formula	Na^+^·C_6_H_13_NaO_9_S^−^
*M*_r_	284.21
Crystal system, space group	Monoclinic, *P*2_1_
Temperature (K)	140
*a*, *b*, *c* (Å)	4.8744 (2), 5.0042 (2), 21.0759 (10)
β (°)	93.867 (4)
*V* (Å^3^)	512.92 (4)
*Z*	2
Radiation type	Mo *K*α
μ (mm^−1^)	0.40
Crystal size (mm)	0.52 × 0.20 × 0.04

Data collection	
Diffractometer	Oxford Diffraction Xcalibur 3/Sapphire3 CCD
Absorption correction	Multi-scan (*CrysAlis PRO*; Agilent, 2014 ▸)
*T*_min_, *T*_max_	0.618, 1.000
No. of measured, independent and observed [*I* > 2σ(*I*)] reflections	9424, 2972, 2926
*R*_int_	0.030
(sin θ/λ)_max_ (Å^−1^)	0.703

Refinement	
*R*[*F* ^2^ > 2σ(*F* ^2^)], *wR*(*F* ^2^), *S*	0.039, 0.093, 1.16
No. of reflections	2972
No. of parameters	206
No. of restraints	7
H-atom treatment	All H-atom parameters refined
Δρ_max_, Δρ_min_ (e Å^−3^)	0.49, −0.45
Absolute structure	Flack *x* determined using 1240 quotients [(*I* ^+^)−(*I* ^−^)]/[(*I* ^+^)+(*I* ^−^)] (Parsons *et al.*, 2013 ▸)
Absolute structure parameter	0.04 (4)

Computer programs: *CrysAlis PRO* (Agilent, 2014 ▸), *SHELXT* (Sheldrick, 2015*a* ▸), *SHELXL2014/7* (Sheldrick, 2015*b* ▸), *ORTEP* (Johnson, 1976 ▸) and *ORTEP-3 for Windows* and *WinGX* (Farrugia, 2012 ▸).

## supplementary crystallographic information

### Crystal data

**Table:**

Na^+^·C_6_H_13_NaO_9_S^−^	*F*(000) = 296
*M**_r_* = 284.21	*D*_x_ = 1.840 Mg m^−^^3^
Monoclinic, *P*2_1_	Mo *K*α radiation, λ = 0.71073 Å
*a* = 4.8744 (2) Å	Cell parameters from 4715 reflections
*b* = 5.0042 (2) Å	θ = 3.9–32.5°
*c* = 21.0759 (10) Å	µ = 0.40 mm^−^^1^
β = 93.867 (4)°	*T* = 140 K
*V* = 512.92 (4) Å^3^	Plate, colourless
*Z* = 2	0.52 × 0.20 × 0.04 mm

### Data collection

**Table:**

Oxford Diffraction Xcalibur 3/Sapphire3 CCD diffractometer	2972 independent reflections
Radiation source: Enhance (Mo) X-ray Source	2926 reflections with *I* > 2σ(*I*)
Graphite monochromator	*R*_int_ = 0.030
Detector resolution: 16.0050 pixels mm^-1^	θ_max_ = 30.0°, θ_min_ = 3.9°
Thin slice φ and ω scans	*h* = −6→6
Absorption correction: multi-scan (CrysAlis PRO; Agilent, 2014)	*k* = −7→7
*T*_min_ = 0.618, *T*_max_ = 1.000	*l* = −29→29
9424 measured reflections	

### Refinement

**Table:**

Refinement on *F*^2^	Hydrogen site location: difference Fourier map
Least-squares matrix: full	All H-atom parameters refined
*R*[*F*^2^ > 2σ(*F*^2^)] = 0.039	*w* = 1/[σ^2^(*F*_o_^2^) + (0.0295*P*)^2^ + 0.694*P*] where *P* = (*F*_o_^2^ + 2*F*_c_^2^)/3
*wR*(*F*^2^) = 0.093	(Δ/σ)_max_ < 0.001
*S* = 1.16	Δρ_max_ = 0.49 e Å^−^^3^
2972 reflections	Δρ_min_ = −0.44 e Å^−^^3^
206 parameters	Absolute structure: Flack *x* determined using 1240 quotients [(I+)-(I-)]/[(I+)+(I-)] (Parsons *et al.*, 2013)
7 restraints	Absolute structure parameter: 0.04 (4)
Primary atom site location: dual	

### Special details

**Table:**

Geometry. All esds (except the esd in the dihedral angle between two l.s. planes) are estimated using the full covariance matrix. The cell esds are taken into account individually in the estimation of esds in distances, angles and torsion angles; correlations between esds in cell parameters are only used when they are defined by crystal symmetry. An approximate (isotropic) treatment of cell esds is used for estimating esds involving l.s. planes.

### Fractional atomic coordinates and isotropic or equivalent isotropic displacement parameters (Å^2^)

**Table:**

	*x*	*y*	*z*	*U*_iso_*/*U*_eq_	
C1	0.5658 (7)	0.4844 (7)	0.83763 (16)	0.0099 (6)	
O1	0.4257 (5)	0.2507 (5)	0.85501 (12)	0.0124 (5)	
C2	0.7379 (7)	0.4250 (7)	0.78081 (16)	0.0092 (6)	
O2	0.9236 (5)	0.2083 (5)	0.79422 (12)	0.0112 (5)	
C3	0.5507 (7)	0.3570 (7)	0.72169 (16)	0.0103 (6)	
O3	0.3628 (5)	0.5738 (6)	0.70848 (11)	0.0124 (5)	
C4	0.7176 (7)	0.2973 (7)	0.66432 (16)	0.0106 (6)	
O4	0.8619 (5)	0.5330 (5)	0.64719 (12)	0.0132 (5)	
C5	0.5343 (7)	0.2160 (7)	0.60581 (16)	0.0121 (6)	
O5	0.3884 (5)	−0.0177 (5)	0.62226 (13)	0.0149 (5)	
C6	0.6989 (8)	0.1565 (7)	0.54832 (17)	0.0142 (7)	
O6	0.8875 (5)	−0.0609 (5)	0.55969 (13)	0.0150 (5)	
S1	0.77100 (15)	0.60171 (15)	0.90636 (4)	0.00914 (16)	
O11	0.5782 (5)	0.7211 (6)	0.94817 (12)	0.0139 (5)	
O12	0.9110 (5)	0.3741 (5)	0.93700 (12)	0.0142 (5)	
O13	0.9575 (5)	0.7966 (5)	0.88046 (12)	0.0125 (5)	
Na	0.2652 (3)	0.0627 (3)	0.94976 (6)	0.0129 (3)	
H1	0.436 (10)	0.615 (13)	0.825 (2)	0.024 (13)*	
H2	0.832 (11)	0.605 (16)	0.776 (3)	0.037 (15)*	
H3	0.412 (9)	0.198 (9)	0.732 (2)	0.007 (10)*	
H4	0.839 (8)	0.167 (8)	0.6749 (19)	0.002 (9)*	
H5	0.377 (10)	0.363 (11)	0.595 (2)	0.020 (13)*	
H61	0.800 (9)	0.326 (9)	0.5382 (19)	0.004 (10)*	
H62	0.588 (9)	0.122 (11)	0.511 (2)	0.015 (11)*	
H1O	0.281 (8)	0.275 (15)	0.834 (3)	0.045 (18)*	
H2O	0.857 (10)	0.118 (11)	0.821 (2)	0.025 (13)*	
H3O	0.458 (14)	0.672 (14)	0.689 (3)	0.08 (3)*	
H4O	1.016 (7)	0.543 (16)	0.666 (3)	0.05 (2)*	
H5O	0.246 (8)	−0.013 (12)	0.601 (2)	0.037 (17)*	
H6O	0.829 (11)	−0.166 (10)	0.585 (2)	0.033 (16)*	

### Atomic displacement parameters (Å^2^)

**Table:**

	*U*^11^	*U*^22^	*U*^33^	*U*^12^	*U*^13^	*U*^23^
C1	0.0066 (14)	0.0104 (15)	0.0125 (15)	−0.0002 (11)	−0.0005 (11)	0.0021 (11)
O1	0.0092 (11)	0.0132 (12)	0.0146 (12)	−0.0038 (9)	0.0000 (9)	0.0016 (9)
C2	0.0068 (13)	0.0078 (14)	0.0130 (14)	−0.0009 (11)	−0.0001 (11)	0.0005 (11)
O2	0.0077 (11)	0.0113 (11)	0.0145 (11)	0.0026 (9)	−0.0005 (9)	0.0014 (9)
C3	0.0094 (14)	0.0106 (15)	0.0108 (14)	−0.0007 (11)	−0.0013 (11)	−0.0001 (11)
O3	0.0094 (10)	0.0124 (12)	0.0152 (11)	0.0009 (10)	−0.0010 (8)	0.0033 (10)
C4	0.0090 (14)	0.0105 (15)	0.0122 (14)	0.0013 (12)	0.0005 (11)	0.0017 (11)
O4	0.0086 (11)	0.0134 (12)	0.0173 (12)	−0.0033 (9)	−0.0019 (9)	0.0022 (9)
C5	0.0120 (15)	0.0113 (15)	0.0129 (15)	−0.0011 (12)	−0.0006 (12)	0.0012 (12)
O5	0.0117 (12)	0.0154 (13)	0.0171 (12)	−0.0043 (10)	−0.0023 (9)	0.0032 (10)
C6	0.0164 (16)	0.0139 (18)	0.0118 (14)	0.0017 (12)	−0.0016 (12)	0.0001 (11)
O6	0.0133 (12)	0.0151 (14)	0.0167 (13)	0.0004 (10)	0.0024 (9)	0.0002 (10)
S1	0.0081 (3)	0.0080 (3)	0.0112 (3)	0.0010 (3)	−0.0003 (2)	−0.0002 (3)
O11	0.0138 (12)	0.0150 (12)	0.0128 (11)	0.0059 (10)	0.0007 (9)	−0.0018 (10)
O12	0.0150 (12)	0.0093 (11)	0.0176 (12)	0.0038 (9)	−0.0033 (9)	0.0012 (9)
O13	0.0104 (11)	0.0100 (11)	0.0171 (12)	−0.0031 (9)	−0.0002 (9)	0.0000 (9)
Na	0.0122 (6)	0.0123 (7)	0.0138 (6)	0.0036 (5)	−0.0006 (5)	−0.0009 (5)

### Geometric parameters (Å, º)

**Table:**

Na—O11^i^	2.293 (3)	O4—H4O	0.82 (3)
Na—O12^ii^	2.328 (3)	C5—O5	1.424 (4)
Na—O11^iii^	2.370 (3)	C5—C6	1.527 (5)
Na—O1	2.386 (3)	C5—H5	1.08 (5)
Na—O13^iv^	2.421 (3)	O5—H5O	0.80 (3)
Na—O12^iii^	2.757 (3)	C6—O6	1.434 (4)
C1—O1	1.415 (4)	C6—H61	1.01 (4)
C1—C2	1.537 (5)	C6—H62	0.94 (4)
C1—S1	1.802 (3)	O6—H6O	0.81 (3)
C1—H1	0.94 (6)	S1—O12	1.456 (3)
O1—Na	2.386 (3)	S1—O11	1.459 (3)
O1—H1O	0.81 (3)	S1—O13	1.464 (3)
C2—O2	1.429 (4)	S1—Na^v^	3.0557 (16)
C2—C3	1.532 (4)	O11—Na^vi^	2.293 (3)
C2—H2	1.02 (8)	O11—Na^v^	2.370 (3)
O2—H2O	0.81 (3)	O12—Na^vii^	2.328 (3)
C3—O3	1.435 (4)	O12—Na^v^	2.757 (3)
C3—C4	1.532 (5)	O13—Na^viii^	2.421 (3)
C3—H3	1.08 (4)	Na—S1^iii^	3.0557 (16)
O3—H3O	0.81 (3)	Na—Na^v^	3.915 (2)
C4—O4	1.432 (4)	Na—Na^iii^	3.915 (2)
C4—C5	1.529 (5)	Na—H1O	2.66 (6)
C4—H4	0.90 (4)		
			
O1—C1—C2	109.9 (3)	C1—S1—Na^v^	135.30 (11)
O1—C1—S1	108.2 (2)	S1—O11—Na^vi^	140.39 (16)
C2—C1—S1	112.8 (2)	S1—O11—Na^v^	103.31 (14)
O1—C1—H1	109 (3)	Na^vi^—O11—Na^v^	114.20 (11)
C2—C1—H1	109 (3)	S1—O12—Na^vii^	153.71 (18)
S1—C1—H1	109 (3)	S1—O12—Na^v^	87.25 (12)
C1—O1—Na	137.2 (2)	Na^vii^—O12—Na^v^	113.65 (10)
C1—O1—H1O	99 (5)	S1—O13—Na^viii^	121.11 (15)
Na—O1—H1O	101 (5)	O11^i^—Na—O12^ii^	170.53 (12)
O2—C2—C3	109.1 (3)	O11^i^—Na—O11^iii^	95.09 (8)
O2—C2—C1	111.4 (3)	O12^ii^—Na—O11^iii^	94.13 (11)
C3—C2—C1	110.5 (3)	O11^i^—Na—O1	91.47 (10)
O2—C2—H2	114 (3)	O12^ii^—Na—O1	85.52 (10)
C3—C2—H2	111 (3)	O11^iii^—Na—O1	121.61 (11)
C1—C2—H2	101 (3)	O11^i^—Na—O13^iv^	88.09 (10)
C2—O2—H2O	106 (4)	O12^ii^—Na—O13^iv^	82.76 (10)
O3—C3—C4	111.1 (3)	O11^iii^—Na—O13^iv^	151.65 (11)
O3—C3—C2	109.1 (3)	O1—Na—O13^iv^	86.37 (10)
C4—C3—C2	111.5 (3)	O11^i^—Na—O12^iii^	90.22 (10)
O3—C3—H3	101 (2)	O12^ii^—Na—O12^iii^	93.28 (8)
C4—C3—H3	113 (2)	O11^iii^—Na—O12^iii^	55.17 (9)
C2—C3—H3	111 (2)	O1—Na—O12^iii^	176.51 (11)
C3—O3—H3O	100 (6)	O13^iv^—Na—O12^iii^	96.74 (10)
O4—C4—C5	106.6 (3)	O11^i^—Na—S1^iii^	98.35 (8)
O4—C4—C3	109.5 (3)	O12^ii^—Na—S1^iii^	88.84 (8)
C5—C4—C3	112.1 (3)	O11^iii^—Na—S1^iii^	27.68 (7)
O4—C4—H4	109 (3)	O1—Na—S1^iii^	148.15 (9)
C5—C4—H4	110 (3)	O13^iv^—Na—S1^iii^	123.98 (8)
C3—C4—H4	109 (3)	O12^iii^—Na—S1^iii^	28.43 (6)
C4—O4—H4O	112 (5)	O11^i^—Na—Na^v^	96.85 (9)
O5—C5—C6	109.3 (3)	O12^ii^—Na—Na^v^	92.10 (7)
O5—C5—C4	107.3 (3)	O11^iii^—Na—Na^v^	32.29 (7)
C6—C5—C4	112.5 (3)	O1—Na—Na^v^	89.32 (8)
O5—C5—H5	105 (3)	O13^iv^—Na—Na^v^	173.53 (8)
C6—C5—H5	112 (3)	O12^iii^—Na—Na^v^	87.45 (8)
C4—C5—H5	111 (3)	S1^iii^—Na—Na^v^	59.55 (4)
C5—O5—H5O	105 (4)	O11^i^—Na—Na^iii^	33.52 (6)
O6—C6—C5	112.4 (3)	O12^ii^—Na—Na^iii^	153.15 (10)
O6—C6—H61	111 (2)	O11^iii^—Na—Na^iii^	65.29 (9)
C5—C6—H61	107 (2)	O1—Na—Na^iii^	119.46 (9)
O6—C6—H62	109 (3)	O13^iv^—Na—Na^iii^	106.89 (7)
C5—C6—H62	114 (3)	O12^iii^—Na—Na^iii^	61.18 (7)
H61—C6—H62	104 (4)	S1^iii^—Na—Na^iii^	64.84 (4)
C6—O6—H6O	111 (4)	Na^v^—Na—Na^iii^	79.45 (5)
O12—S1—O11	110.74 (16)	O11^i^—Na—H1O	102.9 (13)
O12—S1—O13	113.71 (16)	O12^ii^—Na—H1O	72.2 (12)
O11—S1—O13	113.22 (16)	O11^iii^—Na—H1O	132.0 (16)
O12—S1—C1	108.65 (16)	O1—Na—H1O	17.4 (8)
O11—S1—C1	105.88 (16)	O13^iv^—Na—H1O	74.0 (14)
O13—S1—C1	104.03 (15)	O12^iii^—Na—H1O	163.4 (9)
O12—S1—Na^v^	64.32 (11)	S1^iii^—Na—H1O	152.7 (17)
O11—S1—Na^v^	49.00 (11)	Na^v^—Na—H1O	100.8 (14)
O13—S1—Na^v^	119.44 (11)	Na^iii^—Na—H1O	134.2 (11)
			
C2—C1—O1—Na	150.0 (2)	C2—C1—S1—O11	163.8 (2)
S1—C1—O1—Na	26.4 (4)	O1—C1—S1—O13	166.0 (2)
O1—C1—C2—O2	−55.8 (3)	C2—C1—S1—O13	44.2 (3)
S1—C1—C2—O2	65.0 (3)	O1—C1—S1—Na^v^	−27.4 (3)
O1—C1—C2—C3	65.6 (3)	C2—C1—S1—Na^v^	−149.19 (18)
S1—C1—C2—C3	−173.6 (2)	O12—S1—O11—Na^vi^	−179.4 (2)
O2—C2—C3—O3	179.6 (3)	O13—S1—O11—Na^vi^	51.6 (3)
C1—C2—C3—O3	56.8 (3)	C1—S1—O11—Na^vi^	−61.8 (3)
O2—C2—C3—C4	−57.3 (3)	Na^v^—S1—O11—Na^vi^	161.2 (3)
C1—C2—C3—C4	179.9 (3)	O12—S1—O11—Na^v^	19.44 (19)
O3—C3—C4—O4	57.3 (3)	O13—S1—O11—Na^v^	−109.62 (15)
C2—C3—C4—O4	−64.6 (3)	C1—S1—O11—Na^v^	137.03 (14)
O3—C3—C4—C5	−60.8 (4)	O11—S1—O12—Na^vii^	−160.2 (4)
C2—C3—C4—C5	177.2 (3)	O13—S1—O12—Na^vii^	−31.4 (4)
O4—C4—C5—O5	−179.7 (3)	C1—S1—O12—Na^vii^	83.9 (4)
C3—C4—C5—O5	−59.9 (4)	Na^v^—S1—O12—Na^vii^	−144.0 (4)
O4—C4—C5—C6	60.0 (4)	O11—S1—O12—Na^v^	−16.19 (16)
C3—C4—C5—C6	179.9 (3)	O13—S1—O12—Na^v^	112.62 (14)
O5—C5—C6—O6	−57.8 (4)	C1—S1—O12—Na^v^	−132.07 (12)
C4—C5—C6—O6	61.2 (4)	O12—S1—O13—Na^viii^	−67.7 (2)
O1—C1—S1—O12	44.6 (3)	O11—S1—O13—Na^viii^	59.9 (2)
C2—C1—S1—O12	−77.2 (3)	C1—S1—O13—Na^viii^	174.31 (16)
O1—C1—S1—O11	−74.4 (3)	Na^v^—S1—O13—Na^viii^	5.1 (2)

Symmetry codes: (i) *x*, *y*−1, *z*; (ii) *x*−1, *y*, *z*; (iii) −*x*+1, *y*−1/2, −*z*+2; (iv) *x*−1, *y*−1, *z*; (v) −*x*+1, *y*+1/2, −*z*+2; (vi) *x*, *y*+1, *z*; (vii) *x*+1, *y*, *z*; (viii) *x*+1, *y*+1, *z*.

### Hydrogen-bond geometry (Å, º)

**Table:**

*D*—H···*A*	*D*—H	H···*A*	*D*···*A*	*D*—H···*A*
O1—H1*O*···O2^ii^	0.81 (3)	1.92 (4)	2.693 (4)	160 (8)
O2—H2*O*···O1	0.81 (3)	2.36 (5)	2.828 (3)	118 (5)
O2—H2*O*···O13^i^	0.81 (3)	2.08 (4)	2.745 (4)	140 (5)
O3—H3*O*···O4	0.81 (3)	2.32 (7)	2.838 (3)	123 (7)
O3—H3*O*···O5^vi^	0.81 (3)	2.11 (6)	2.743 (4)	136 (7)
O4—H4*O*···O3^vii^	0.82 (3)	1.87 (3)	2.692 (3)	179 (8)
O5—H5*O*···O6^ii^	0.80 (3)	1.91 (3)	2.704 (4)	169 (6)
O6—H6*O*···O4^i^	0.81 (3)	2.00 (4)	2.753 (4)	154 (6)
O6—H6*O*···O5	0.81 (3)	2.45 (6)	2.853 (4)	112 (5)

Symmetry codes: (i) *x*, *y*−1, *z*; (ii) *x*−1, *y*, *z*; (vi) *x*, *y*+1, *z*; (vii) *x*+1, *y*, *z*.

## References

[bb1] Agilent (2014). *CrysAlis PRO.* Agilent Technologies Ltd, Yarnton, England.

[bb2] Cole, E. R., Craig, D. C., Fitzpatrick, L. J., Hibbert, D. B. & Stevens, J. D. (2001). *Carbohydr. Res.* **335**, 1–10. 10.1016/s0008-6215(01)00206-3 11553349

[bb3] Farrugia, L. J. (2012). *J. Appl. Cryst.* **45**, 849–854.

[bb4] Haines, A. H. & Hughes, D. L. (2010). *Carbohydr. Res.* **345**, 2705–2708. 10.1016/j.carres.2010.09.019 20971450

[bb5] Haines, A. H. & Hughes, D. L. (2012). *Acta Cryst.* E**68**, m377–m378. 10.1107/S1600536812007210 PMC3343795 22589769

[bb6] Haines, A. H. & Hughes, D. L. (2014). *Acta Cryst.* E**70**, 406–409. 10.1107/S1600536814022685 PMC4257350 25484759

[bb7] Haines, A. H. & Hughes, D. L. (2015). *Acta Cryst.* E**71**, 993–996. 10.1107/S2056989015014139 PMC4571375 26396774

[bb8] Haines, A. H. & Hughes, D. L. (2016). *Acta Cryst.* E**72**, 628–631. 10.1107/S2056989016005375 PMC4908541 27308005

[bb9] Johnson, C. K. (1976). *ORTEPII.* Report ORNL-5138. Oak Ridge National Laboratory, Tennessee, USA.

[bb10] Kissane, M. G., Frank, S. A., Rener, G. A., Ley, C. P., Alt, C. A., Stroud, P. A., Vaid, R. K., Boini, S. K., McKee, L. A., Vicenzi, J. T. & Stephenson, G. A. (2013). *Tetrahedron Lett.* **54**, 6587–6591.

[bb11] Parsons, S., Flack, H. D. & Wagner, T. (2013). *Acta Cryst.* B**69**, 249–259. 10.1107/S2052519213010014 PMC3661305 23719469

[bb12] Sheldrick, G. M. (2015*a*). *Acta Cryst.* A**71**, 3–8.

[bb13] Sheldrick, G. M. (2015*b*). *Acta Cryst.* C**71**, 3–8.

